# Promoting Innovative Behavior in Employees: The Mechanism of Leader Psychological Capital

**DOI:** 10.3389/fpsyg.2020.598090

**Published:** 2021-01-12

**Authors:** Yanfei Wang, Yi Chen, Yu Zhu

**Affiliations:** ^1^School of Business Administration, South China University of Technology, Guangzhou, China; ^2^Xingjian College of Science and Liberal Arts, Guangxi University, Nanning, China; ^3^School of Management, Jinan University, Guangzhou, China

**Keywords:** psychological capital, leader psychological capital, psychological safety, growth need strength, innovative behavior, conservation of resources theory

## Abstract

The study reported in this paper analyzed the influence of leader psychological capital (PsyCap) on employees’ innovative behavior and the roles of psychological safety and growth need strength (GNS) in this process within the context of positive psychology theory and conservation of resources theory. Three stages of questionnaire surveys were administered to 81 enterprise leaders and their 342 direct subordinates in South China to test our theoretical model. The results showed that leader PsyCap had significant and positive effects on employee innovative behavior, psychological safety had a partially mediating effect, and GNS positively moderated the relationship between psychological safety and innovative behavior. The results revealed the mechanism of PsyCap and external boundary conditions of the influence of leader PsyCap on employee innovative behavior. The study expands the research results of leader PsyCap theory and also provides guidance on how enterprises manage employees’ innovative behavior.

## Introduction

Innovative behavior is effective for an organization to adapt to environmental changes and maintain its competitiveness (Devloo et al., 2014; Parahoo et al., 2017). Employee innovation plays a key role in providing continuous competitive advantage to the organization (Ghosh, 2010; Turró et al., 2014; Odoardi et al., 2015; Shin et al., 2017). Many literature concern with employee innovation, and these reviews identified that employee psychological capital has a positive effect on employee innovation (Liang and Li, 2016; Fang et al., 2019; Tang et al., 2019; Tsegaye et al., 2020), but few of these studies focus on the relationship between leader psychological capital and employee innovation (Peterson et al., 2009; Li et al., 2020). Positive psychology posits that leader psychological capital (PsyCap) is a positive psychological resource. In addition to an active attitude toward their work and high working performance, leaders with a high PsyCap level are enthusiastic at work and set an example for their followers (Walumbwa et al., 2010). The literature shows that the innovation ability of employees is influenced by their leaders (Zubair et al., 2015; Liu et al., 2017; Harbi et al., 2019; Su et al., 2019), and research on how to improve employee innovation by leader psychological capital is growing (Mohamad et al., 2019). However, knowledge on the level of leader psychological capital affected employees’ innovative behavior remains ambiguous (Li et al., 2020). Therefore, this study explored the effect of leader PsyCap on innovative behavior in employees.

Innovative behavior, which is often associated with uncertain outcomes, is considerably risky (Janssen, 2003; Hon, 2013; Hon and Lu, 2015; Hon and Lui, 2016; Mumtaz and Parahoo, 2019). For example, an employee with creative ideas may challenge the given organizational policies, working methods, and mission (Hon, 2012), but his or her supervisor and colleagues might refuse to these creative minds to change in order to avoid insecurity and stress due to the change (Jones, 2001; Janssen, 2003). The presentation of new ideas may challenge the established way of conduct or infringe upon the vested interest of other members of the organization (Edmondson et al., 2001; Detert and Burris, 2007). Moreover, trying new ways in the workplace may end up with failure, which causes the negative view on the relevant individuals. Once risks are involved, the lack of psychological safety will prevent employees from proposing innovative ideas and more favorable methods of working (Newman et al., 2017). Employees are motivated to be innovative only when they perceive that their interpersonal relationships will remain intact after proposing an innovative project (Edmondson, 1999). Psychological safety is the perception and experience of a high level of interpersonal trust (Hu et al., 2018). Accordingly, this study hypothesized that psychological safety is an antecedent of employee innovative behavior. Leadership largely affects employees’ psychological safety (Edmondson and Lei, 2014). Empirical studies evidenced that openness of the management and a good relationship between leaders and subordinates will improve the psychological safety of the followers (Edmondson, 2004; Li et al., 2015). Leaders with high psychological capital will contribute to effective interpersonal relationship, settlement of misunderstanding and conflict, and a healthy working environment (Chen et al., 2017). When leaders exhibit positive psychological characteristics (i.e., hope, optimism, self-efficacy, and flexibility), the subordinates are likely to believe that their leaders have strong leadership and have trust in them (Norman et al., 2010), that is, the employees’ trust in their leaders is positively correlated with leader PsyCap level. Therefore, leader PsyCap may indirectly affect innovative behavior through the mediating effect of psychological safety among employees during the entire process.

Engaging in innovative behavior requires employees to consume various resources, including time, effort, and emotional labor. A study by Graen et al. argued that a high level of growth need strength (GNS) is a prerequisite for motivating employees to finish complex and challenging tasks (Graen et al., 1986). Individuals with high GNS have strong initiative, emphasize personal growth, development, and achievement, and positively respond to challenging and stimulating tasks (Chae and Choi, 2018), from which they can obtain a higher level of intrinsic motivation (Tiegs et al., 1992). The conservation of resources theory posits that individuals are highly motivated to acquire new resources and avoid resource loss because losing or owning few resources can increase pressure (Halbesleben et al., 2014). Therefore, when encountering innovative work that is highly risky and challenging, individuals with high GNS tend to consider innovation as an opportunity for personal growth, development, and resource acquisition. By contrast, those with low GNS refuse to perform innovative yet adventurous tasks in order to protect their resources. Few scholars have conducted empirical research on GNS and its relationship with innovative behavior (Li et al., 2018). This study introduced GNS as a moderator to analyze its boundary effect on the relationship between leader PsyCap and innovative behavior in employees.

On the basis of positive psychology and conservation of resources theory, this study explored the effect of leader PsyCap on employee innovative behavior, the mediating effect of psychological safety, and the moderating effect of GNS. Despite being rarely studied in academia, leader PsyCap is an essential psychological resource that can be effectively developed in an organization. Moreover, because employee innovation largely affects organizational competitive advantage, the effect of leader PsyCap on such innovation warrants further exploration. The results of this study are expected to diversify and expand leader PsyCap research and provide suggestions for managers on solving problems related to employee innovation from the perspective of leader PsyCap.

## Theory and Hypotheses

### Relationship Between Leader PsyCap and Employee Innovative Behavior

As a critical concept of positive psychology, psychological capital was first introduced and extended to the field of organizational management by Luthans. Psychological capital indicates the psychological state of the employees who show positive organizational behavior composed of four items such as hope, self-efficacy, resilience, and optimism (Luthans, 2012; Wang et al., 2013), the combined initials of the four items being HERO, which displays the positive feature of core psychological capital. Moreover, as an integrated concept, these four elements illustrate that an individual has an optimistic assessment of his or her surroundings and reasonable anticipation of the possibility of success based on the reasonable evaluation of his or her own ability and resource. HERO also shows the psychological feature and behavior tendency of an individual who persists to hit the expected target when facing a difficulty (Grgens-Ekermans and Herbert, 2013). Previous literature evidence that psychological capital can forecast the positive perception, attitude, and initiative behavior such as job satisfaction, organizational commitment, and organizational citizenship behaviors (Avey et al., 2011b; Newman et al., 2014). Leader PsyCap is a critical research subfield of PsyCap. Leader PsyCap refers to a psychological property that motivates leaders to develop a positive mental state, shape positive organizational behaviors, pay attention to themselves and their subordinates, and focus on helping subordinates achieve their full potential, all of which contribute to improved business performance (Jensen and Luthans, 2006). Four characteristics of leader PsyCap, namely, self-efficacy, optimism, resilience, and hope as well as the overall concept of PsyCap facilitate leadership effectiveness, creating opportunities for a team to achieve outstanding performance and other positive work outcomes (Norman et al., 2005, 2010).

Creativity is a concept closely related to innovation; the two terms can be used interchangeably in certain cases (e.g., Amabile, 1988; Martins and Terblanche, 2013; Anderson et al., 2014). It was proposed that creativity and innovation are structurally connected, which are the two consecutive periods in the process of introducing new and improved ways to the job. Hence, in order to reveal a huge innovative organization, creativity and innovation should combine together rather than separate from each other (Hon and Lui, 2016), but there is no consensus among scholars on the specific definition of the two concepts (Anderson et al., 2014). Creativity and innovation are considered to be different and correlated (e.g., Ghosh, 2015; Castañer, 2016). Creativity is referred to as a significant antecedent variable of innovation (Amabile et al., 1996; Heye, 2006; Schilling, 2008). Shilling (2006) proposed that creative thinking is the base of innovation, while innovation is the successful performance of creative thinking. Additionally, individuals with novel and unique thinking are more likely to innovate effectively (Woodman et al., 1993). According to the concept defined by Scott and Bruce (1994), our study refers to innovation to be a course of issues as employee identification, proposing new ideas and solution, and generating a new product, which concedes with a previous study that innovation is the successful performance of creative thinking (Shilling, 2006).

Employee innovative behavior occurs when employees practice their innovative ideas generated in organizational activities, including innovation, technological development, and management procedural changes (Shi, 2012). Sheppard et al. (2010) confirmed that a positive work attitude is a deciding factor affecting individual innovation. Norman et al. (2010) demonstrated that leaders with positive psychological states (i.e., confidence, optimism, hopefulness, and resilience) serve as an example for subordinates. Leaders with a higher level of psychological capital have much higher possibility and driving force for success in addition to setting a more challenging target. They are also more willing to achieve success as well as find solutions to overcome obstacles positively (Peterson et al., 2009; Avey et al., 2011a). Subordinates with high leader PsyCap had significantly enhanced problem-solving skills and creativity than those with low leader PsyCap did (Avey et al., 2011a, 2012). Meanwhile, leaders with a higher level of psychological capital show a more positive attitude toward their followers because they see more opportunity to achieve the goal and show more confidence and support to their followers (Norman et al., 2010; Story et al., 2013). In an empirical study, Zhu and Wang (2011) demonstrated that the entrepreneurs’ optimistic and flexible attitudes allow the employees to experiment with new ideas despite the probability of failure. Therefore, this study assumed that leaders with high PsyCap positively affect their employees’ innovative behavior. Accordingly, this study proposed the following hypothesis:

H1: Leader PsyCap significantly and positively affects employee innovative behavior.

### The Mediating Effect of Psychological Safety

Psychological safety is the belief among employees that they can participate in risky acts in an organization without affecting their image or status (Kahn, 1990). As a perception that one’s actions are safe, psychological safety reduces the employees’ expectation that proposing a new idea will be risky (Liang et al., 2012), enabling them to focus on improvement and find new solutions (Carmeli et al., 2014) rather than worrying about how others will react to their behavior (Frazier et al., 2017). Palanski and Vogelgesang (2011) contended that psychological safety positively predicts employees’ innovative thinking and willingness to engage in risky activities. Therefore, this study expected psychological safety to positively affect employee innovation.

Psychological safety is subject to the individuals and systems of an enterprise, wherein the effect of leaders is the most substantial (May et al., 2004). When leaders are open-minded, available, and easy-going, the employees’ psychological safety will be developed as a result. Leader PsyCap is a critical element to build up effective leader-member relation since it can enhance the charming personality of leaders (Luthans et al., 2007b). Confident leaders believe in his or her ability in the course of management and provide more support for employees (Bandura, 1997). A leader with a high level of hope can come up with various solutions in terms of different situations, attain the group goal, and earn the trust of the members (Snyder and Shorey, 2003). Optimistic team leaders hope for the best for the future; they believe that they can remove the obstacles to achieve the goal with their effort and encourage members as well (Brissette et al., 2002). Resilient leaders are accomplished in guiding members to gain a positive emotion, leading the team to go through the adverse situation and setbacks (Masten and Reed, 2002). To sum up, high PsyCap leaders play a key role in establishing a relationship of faithfulness, harmony, and mutual trust among team members, and the organizational atmosphere of equality, tolerance, and trust helps to improve the employees’ sense of psychological safety (Zeng et al., 2020). Accordingly, this study hypothesized that leader PsyCap positively affects psychological safety among employees and proposed the following hypotheses:

H2a: Leader PsyCap significantly and positively affects psychological safety.
H2b: Psychological safety significantly and positively affects innovative behavior.
H2c: Psychological safety mediates the effect of leader PsyCap on innovative behavior.

### The Moderating Effect of GNS

GNS refers to the degree to which individuals attach importance to personal growth and development opportunities at work (Oldham and Hackman, 2010) and to a person’s motivation for personal achievement and growth as well as a desire for independent thinking and acts (Hackman and Oldham, 1980). The higher a person’s GNS level, the more he or she craves difficult challenges (Nguyen, 2019). Individuals with high GNS “want to learn new things, stretch themselves, and strive to do better in their jobs” (Shalley et al., 2009, p. 489) and are more likely to look for opportunities to expand and demonstrate their innovation (Mumtaz and Parahoo, 2019). In innovative work, GNS is an internal drive that motivates employees (Lin et al., 2016). Without such internal drive, employees are rarely motivated to continue focusing on innovative work (Liu et al., 2016).

The conservation of resources theory proposes that individuals are inclined to obtain, retain, foster, and protect their cherished resource (Hobfoll, 1989; Hobfoll et al., 2018). Individuals with abundant resources are less likely to be affected by resource losses and can obtain resources more easily (Wheeler et al., 2012; Hobfoll et al., 2018). In the case of resource losses, individuals will endeavor to guard the existing resource by resource investment. They will also attain new resource to less net loss deprived from original resource obtained, which leads to resource gain spirals (Hobfoll, 1989; Hobfoll et al., 2018). Halbesleben et al. (2014) defined resource as any item that can be perceived by individuals to aid for accomplishing something. Individuals will evaluate the value of resource from two aspects: one is whether a certain item possesses universal value based on the culture one is cultivated; the other is the matching level between certain items and demand of individuals. As mentioned above, psychological safety positively predicts employees’ intent to risk and creative thinking (Palanski and Vogelgesang, 2011). Consequently, psychological safety can be regarded as a kind of psychological resource matching with innovative work. In addition, growth need strength is identified to be positively related to openness to experience which is individual difference relevant to creativity (De Jong et al., 2001; George and Zhou, 2001), so growth need strength can be regarded as another psychological resource matching with innovative work. In terms of the conservation of resources theory, innovative work can be considered as a context of resource loss. This perceived resource loss will result in either withdrawal behavior of employees to protect their residual resource or employees’ deployment of residual resource to gain more new resource (Kiazad et al., 2014). High-GNS employees are internally motivated to consider innovative yet challenging work as an opportunity to acquire knowledge, pursue self-improvement, fulfill self-growth and development needs, and obtain more resources. Accordingly, if employees possess both high GNS and psychological safety, they will take the initiative to invest resources in innovation to acquire more resources. The success of innovation can enable them to obtain resources in an incremental manner (e.g., acquire more confidence or more positive evaluations from leaders and colleagues). Even if employees with high GNS make mistakes, they regard these mistakes as opportunities for learning and honing their skills. They then actively seek solutions to correct the mistakes. Employees with high GNS continually hold the perception that their self-growth and development needs are met when learning from mistakes, and they exhibit more innovative behaviors. This forms a cycle that continually creates value-added resources.

According to the conservation of resources theory, individuals with less initial resource are prone to suffering from resource loss and weaker ability to gain new resource, and employees with limited resource will take negative action to secure existing resource (Hobfoll, 2011; Xu et al., 2017; Lan et al., 2020). Due to the stress and pressure caused by resource loss, individuals endeavor to avoid resource loss (Halbesleben and Bowler, 2007; Halbesleben, 2010; Halbesleben and Wheeler, 2011). Employees with less GNS perceived that innovative work will not bring about added value in resource, but instead it will cost their resource. Even when they perceive sufficient psychological safety, these employees strive to maintain existing resources and reduce investment in innovative behavior to avoid consuming their own resources. Consequently, employees with low GNS do not or rarely exhibit innovative behavior. Regarding employees with high GNS, innovative behavior can considerably differ in employees with high and low psychological safety. By contrast, in employees with low GNS, those with high and low psychological safety rarely engage in innovation, resulting in few differences in innovation between the two groups. On the basis of the aforementioned assumptions, this study proposed the following hypothesis:

H3a: GNS moderates the relationship between psychological safety and innovative behavior. That is to say, psychological safety affects innovative behavior more in employees with high GNS compared with those with low GNS.

Relevant empirical studies have suggested that the GNS level moderates employee innovation. For example, employee GNS can moderate the relationship between their opportunities for growth and innovation (Graen et al., 1986). Accordingly, this study proposed another hypothesis as follows:

H3b: GNS moderates the mediating effect between leader PsyCap and innovative behavior caused by psychological safety, that is, the mediating effect is stronger among employees with high GNS.

**Figure 1:**
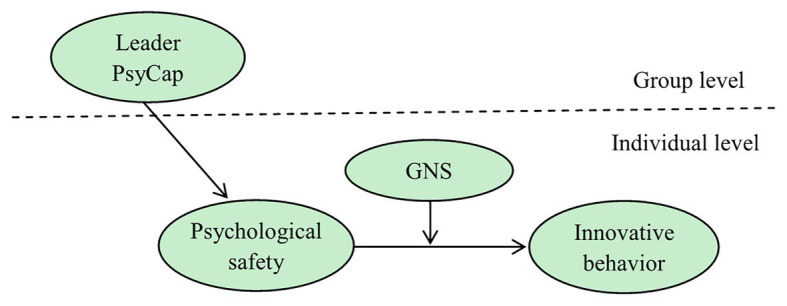
Theoretical framework.

This study established a theoretical model summarizing the aforementioned theoretical analyses and logical inferences (Figure 1).

## Materials and Methods

### Research Samples and Sampling Process

To avoid common-method variance, multisource and multiwave data collection methods were integrated with paired data collection. Employees and their direct supervisors working in South China were recruited as research participants. Specifically, the researchers selected several supervisors who are alumni of a business college, students in a Master of Business Administration program, or students in a training class for top-level managers at a university in southern China. Each supervisor was asked to randomly select at least three subordinates and provide their e-mail addresses. When recording the email address, we mark every supervisor as supervisors1, supervisors2, supervisors3, and so forth. Similarly, we name every subordinate following the corresponding supervisor as subordinate1-1, subordinate1-2, subordinate1-3, etc. We match the data and assure anonymity simultaneously. In total, 96 supervisors and 418 of their direct subordinates were selected to be participants in this study. Before the investigation, the researchers sent emails to all participants, informing them about the research purpose and process and assuring them of anonymity and confidentiality. A questionnaire survey was conducted after these participants granted their consent to participate. Data were collected in three stages. In the first stage, a leader PsyCap questionnaire (Questionnaire A) was distributed to the supervisors. In the second stage, 2 to 3 months after the Questionnaire A responses were collected, a questionnaire measuring psychological safety and GNS was distributed to the subordinates (Questionnaire B). Finally, the supervisors were asked to complete a questionnaire on their subordinates’ innovative behavior (Questionnaire C) 2–3 months after the Questionnaire B responses were collected. After excluding incomplete and unmatched responses and those failing to meet the questionnaire requirements, 81 and 342 valid responses were collected from the supervisors and their subordinates, respectively. The supervisor-subordinate ratio was 1:4.22, with response rates reaching 84.4 and 81.8% for the supervisors and the subordinates, respectively.

The sample consisted of a total of 81 leaders (58.0% males; 42.0% females). Leaders with the following characteristics comprised the largest percentage proportions in the sample: 31–35 years old (39.5%), undergraduate level of education (49.4%), 8 to 10 years of work experience (39.5%), and middle managers (43.2%). There was a total of 342 subordinate participants (47.1% males; 52.9% females). Employees aged ≤25, 26–30, 31–35, 36–40, and ≥ 41 years accounted for 24.6, 35.7, 21.3, 9.6, and 8.8% of the sample, respectively. Regarding their educational attainment, 9.9, 26.9, 53.2, and 9.9% of the subordinates had a high school degree or less, a vocational college diploma, a bachelor’s degree, and a master’s degree or higher, respectively. Most employees had worked for 2–4 years (27.8%), followed by their counterparts who had worked for >10 years (24.6%), <2 years (19.0%), 5–7 years (17.0%), and 8–10 years (11.7%). Most of the subordinates (23.4%) had worked with their supervisors for less than 1 year, followed by those who had worked for 1 to 2 years (22.2%), >5 years (21.9%), 2 to 3 years (18.1%), and 3–5 years (14.3%) with their supervisors.

### Variable Measurement

This study selected scales published in well-known journals to ensure their validity and reliability. A standard translation/back-translation procedure was adopted for the English scales. Leader PsyCap was measured using the 24-item scale developed by Luthans et al. (2007a) that divides leader PsyCap into four dimensions: hope, optimism, self-efficacy, and resilience (Cronbach’s *α* = 0.89). Psychological safety was measured using the one-dimensional scale designed by Edmondson (1999) with seven items (Cronbach’s *α* = 0.78). GNS was measured using the Hackman-Oldham scale (1980) including six items (Cronbach’s *α* = 0.84). The eight-item one-dimensional scale developed by Wang and Zhu (2012) was adopted to measure innovative behavior (Cronbach’s *α* = 0.82). All the items in these scales were rated using a five-point Likert scale (1 = strongly disagree; 5 = strongly agree).

### Discriminant Validity of Variables

In Harman’s single-factor test, the first unrotated factor explained 23.11% of covariance, which was less than the 40% of the threshold, indicating no serious common-method bias in the research data. Furthermore, in a confirmatory factor analysis of these data, the discriminant validity of each variable was identified using model comparison. Table 1 indicates that the four-factor model exhibited optimal fit, demonstrating desirable discriminant validity among the constructs in this study.

**Table 1:** Confirmatory factor analysis of discriminant construct validity between variables.

Model		*χ*^2^	*df*	*χ*^2^/*df*	CFI	TLI	RMSEA	SRMR
Single-factor^a^		2,154.507	709	3.039	0.755	0.716	0.077	0.080
Two-factor^b^		1,723.769	708	2.435	0.828	0.800	0.065	0.071
Three-factor^c^		1,369.572	706	1.940	0.887	0.869	0.052	0.062
Four-factor^d^		1,194.879	702	1.702	0.916	0.902	0.045	0.059

a Leader PsyCap + psychological safety + GNS + innovative behavior.

b Leader PsyCap + psychological safety + GNS; innovative behavior.

c Leader PsyCap + psychological safety; GNS; innovative behavior.

d Leader PsyCap; psychological safety; GNS; innovative behavior.

### Strategies for Statistical Analysis

Leader PsyCap was regarded as a group-level variable in the theoretical model, whereas psychological safety, innovative behavior, and GNS were individual-level variables. The study used Mplus7.4 (Los Angeles, CA, USA) to develop multilevel structural equations to test the theoretical model. Because supervisors self-rated their leader PsyCap, data aggregation was not required. However, before statistical analysis, the interclass difference between outcome variables of innovative behavior required verification to assess the necessity of cross-level analysis. The ICC(1) of innovative behavior was 0.39, which exceeded 0.12. This indicated that 39% of employee innovation variance was caused by - class differences, thereby necessitating a cross-level analysis for hypothesis verification.

## Results

### Descriptive Statistics

Table 2 presents the descriptive statistics of all variables. The coefficients between leader PsyCap and psychological safety (*r* = 0.463; *p* < 0.01) and between leader PsyCap and innovative behavior (*r* = 0.440; *p* < 0.0) reached significance. The coefficient between psychological safety and innovative behavior (*r* = 0.302; *p* < 0.01) was also significant, demonstrating that the variables of interest were closely related and thereby providing the foundation for subsequent hypothesis verification.

**Table 2:** Descriptive statistics and correlations between all variables (*N* = 342).

Variable	1	2	3	4	5	6	7	8	9
Subordinate sex									
Subordinate age	−0.04								
Subordinate educational attainment	−0.024	−0.028							
Subordinate work experience (years)	0	0.731^**^	−0.088						
Subordinate’s years of service with the supervisor	0.021	0.410^**^	−0.06	0.553^**^					
Leader PsyCap	0.033	0.004	0.088	0.065	0.036	**−0.89**			
Psychological safety	−0.066	0.009	0.021	0.065	−0.048	0.463^**^	**−0.78**		
GNS	0.005	0.012	0.025	−0.043	−0.018	0.304^**^	0.09	**−0.84**	
Innovative behavior	−0.039	−0.029	0.073	−0.003	0.037	0.440^**^	0.302^**^	0.230^**^	**−0.82**
Mean	1.57	2.49	2.68	2.96	2.96	3.975	3.663	3.937	3.538
Standard deviation	0.758	1.365	0.992	1.498	1.612	0.413	0.552	0.671	0.592

** *p* < 0.01.

The bold numbers refer to the internal consistency reliability coefficient of the corresponding scales.

### Hypothesis Verification

**Figure 2:**
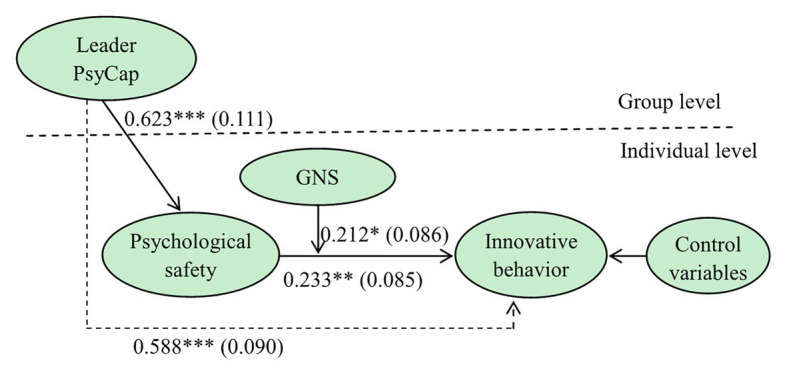
Analysis of the multilevel structural equation model.

To verify the aforementioned hypotheses, this study used Mplus7.4 to develop a multilevel structural equation model (Figure 2). An analysis of this model identified the path coefficients and standard deviations (SDs) between all variables. Specifically, leader PsyCap positively affected psychological safety (*β* = 0.623; *p* < 0.001), supporting H2a. Similarly, psychological safety positively affected innovative behavior (*β* = 0.233; *p* < 0.01), supporting H2b. The verification of H2a and H2b supported H2c. The direct effect of leader PsyCap on innovative behavior was also significant (*β* = 0.588; *p* < 0.001), indicating that psychological safety partially mediated the relationship between leader PsyCap and innovative behavior. Finally, GNS positively moderated the relationship between psychological safety and innovative behavior (*β* = 0.212; *p* < 0.05), supporting H3a.

**Figure 3:**
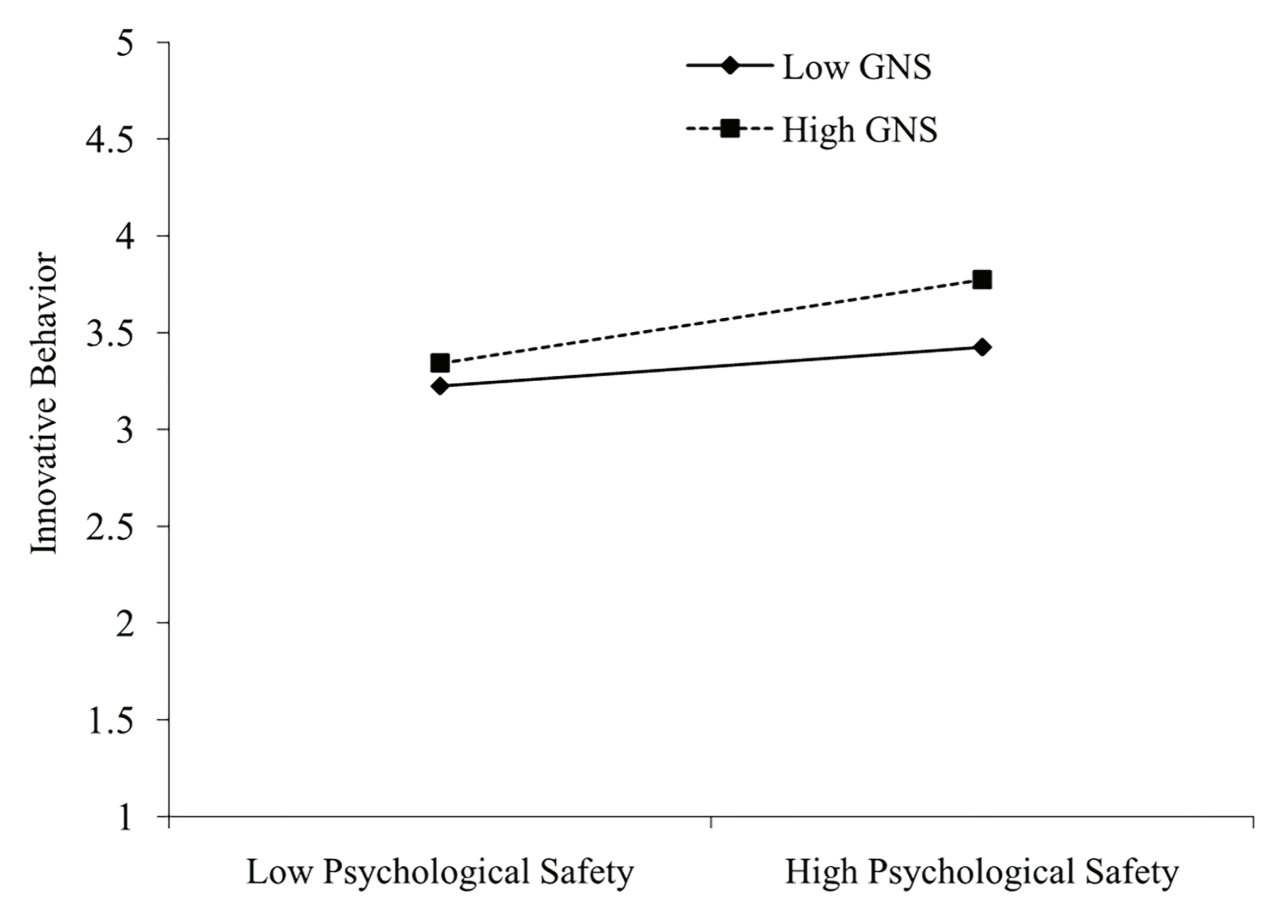
Moderating effect of growth need strength on the relationship between psychological safety and innovative behavior.

To clearly explain the moderating effect of GNS on the relationship between psychological safety and innovative behavior, we plotted simple slopes for the relationship between psychological safety and innovative behavior at high (mean + SD) and low (mean-SD) levels of GNS (Figure 3). Figure 3 illustrates that, compared with employees with low GNS, the regression slope between innovative behavior and psychological safety is greater for employees with high GNS. Specifically, when GNS was high, the positive effect of psychological safety on innovative behavior was significant (*β* = 0.39; *p* < 0.001). By contrast, when GNS was low, the positive effect of psychological safety on innovative behavior was nonsignificant (*β* = 0.18; *p* > 0.05), thereby supporting H3a.

Because a cross-level analysis was used to verify the proposed hypotheses, the researchers simulated the confidence interval by using the Monte Carlo approaches, the results of which were used to verify the total, mediating, and moderated mediating effects. As presented in Table 3, the mediating role of psychological safety in the relationship between leader PsyCap and innovative behavior was 0.150 (95% confidence interval, CI: 0.04–0.28), further supporting H2c. The direct effect of leader PsyCap on innovative behavior was 0.734 (95% CI: 0.54–0.93); thus, H1 was supported. In a high-GNS scenario, the indirect effect of leader PsyCap on innovative behavior through psychological safety was 0.376 (95% CI: 0.12–0.37), whereas the effect was 0.091 in a low-GNS scenario (95% CI: −0.08–0.22), resulting in a difference of 0.285 between the two values (95% CI: 0.04–0.32). Therefore, the indirect effect of leader PsyCap on innovative behavior *via* psychological safety was moderated by GNS. The higher the GNS, the stronger the effect of leader PsyCap on innovative behavior through psychological safety, thereby verifying H3b.

**Table 3:** Moderated mediation results using Monte Carlo methods.

Outcome variable	Psychological safety	Effect value	Standard deviation	Lower limit	Upper limit
Mediation path	–	0.150	0.061	0.04	0.28
	High GNS	0.376	0.082	0.12	0.37
	Low GNS	0.091	0.119	−0.08	0.22
	Difference between high- and low-GNS results	0.285	0.115	0.04	0.32
Total path	–	0.734	0.099	0.54	0.93

## Discussion

On the basis of the conservation of resources theory, this study developed a multilevel linear model to analyze the effect of leader PsyCap on innovative behavior. The study findings were as follows: (1) Leader PsyCap positively affected subordinate innovative behavior. Specifically, leaders with high PsyCap are confident about future development, adept at eliciting positive emotions in employees, and can support their employees with empathy. Therefore, supervisors with high leader PsyCap can positively affect employee innovation; (2) Psychological safety partially mediated the effect of leader PsyCap on innovative behavior, suggesting that leader PsyCap affects innovative behavior through its effect on risk perception among employees; (3) Regarding the moderating effect of GNS on the relationship between psychological safety and innovative behavior, this study demonstrated that innovative behavior was consistently rare among employees with low GNS regardless of psychological safety in the workplace. By contrast, when employees with high GNS perceived that innovation failure would not affect their status or image, they actively engaged in innovative work.

### Theoretical Implications

This study investigated the impact of leader PsyCap on innovative behavior, which contributes to leadership research, especially leader PsyCap research. Most of the previous studies highlight on the diverse style of leadership, such as humble leadership, coaching leadership, and so on. Besides this, research on the relationship between PsyCap and employees’ innovation are merely limited to employees’ PsyCap (Bruccoleri and Riccobono, 2018). Considering leader PsyCap as an antecedent, this study examined the positive impact of leader PsyCap on employees’ innovative behavior through the effect of psychological safety, elaborating the mechanism of leader PsyCap affecting employee innovation and diversifying and filling gaps in leader PsyCap research.

The researchers explored the mediation effect of subordinate psychological safety in the relationship between leader PsyCap and innovative behavior. Previous empirical studies verified that psychological safety was a vital cognitive process which links leaders and followers (Hirak et al., 2012; Zhu and Zhang, 2019), but existing literature over the research on antecedents of psychological safety is included in an area that involves the following styles: transformational leadership (i.e., Detert and Burris, 2007; Nemanich and Vera, 2009), ethical leadership (i.e., Walumbwa and Schaubroeck, 2009; Sağnak, 2017; Hu et al., 2018; Men et al., 2018), servant leadership (i.e., Schaubroeck et al., 2011; Chughtai, 2016), empowering leadership (i.e., Jada and Mukhopadhyay, 2018), humble leadership (i.e., Wang et al., 2018a,b), and leader-member exchange (Hu et al., 2018; Opoku et al., 2020). Moreover, existing studies over the result of psychological safety mainly focus on information sharing (Siemsen et al., 2009; Bunderson and Boumgarden, 2010), voice behavior (i.e., Chughtai, 2016; Sağnak, 2017; Hu et al., 2018; Jada and Mukhopadhyay, 2018; Opoku et al., 2020; Song et al., 2020), creativity (Wang et al., 2018a,b), and task performance (Baer and Frese, 2003; Schaubroeck et al., 2011). Scarce literature discuss the mediating effect of psychological safety in the relationship between leader PsyCap and employees’ innovative behavior. Frazier et al. (2017) suggested that examining leadership impact on psychological safety from various prospect will contribute to the research on psychological safety and leadership. This finding provides insight for leader PsyCap theories, which consist of findings by Frazier et al. (2017), thereby expanding research on the mechanism of psychological safety and supplementing current research on psychological safety and leadership.

This study revealed and confirmed the moderating effect of GNS on the relationship between psychological safety and innovative behavior. Research on GNS originated from the job characteristics model. However, current research regard GNS a specific personality trait and explore GNS effect on employees’ positive behavior beyond the model (i.e., Shalley et al., 2009; Lin et al., 2016; Wang et al., 2018b). Similarly, this study considers GNS a positive personality trait, introducing GNS to the research framework for employee innovation and examining the moderating effect of GNS on the effect of leader PsyCap on innovative behavior through psychological safety on the basis of the conversation of resource theory. Individuals with high GNS proactively invested resources in innovation when their psychological safety was guaranteed. By contrast, in scenarios with high GNS that lacked safety, low GNS with sufficient safety, and low GNS that lacked safety, individuals proactively reduced resource-consuming innovation to protect their resources. Consequently, innovative behavior in these three scenarios was rare. Newman et al. (2017) suggested that alternate theories, such as the conservation of resources theory, should be adopted to explain how psychological safety, generated by resource acquisition at work, encourages employees to invest their resources in learning, self-growth, and self-development. The theoretical analyses and empirical results of this study responded to this suggestion and applied the conservation of resources theory.

### Practical Implications

Organizations should emphasize the development and management of leader PsyCap. The path of leader PsyCap affecting innovative behavior through employee psychological safety demonstrated that the positive effect of leader PsyCap on innovative behavior should be highlighted in organizational management. Firstly, organizations should pay more attention to developing and improving leader PsyCap. Due to the state-like and exploitable traits of PsyCap (Luthans et al., 2010), organizations should purposely arrange for managers to receive specific training and increase leader PsyCap by a series of practice in accordance with the four dimensions of construct of PsyCap. PsyCap can also be rapidly improved by building a positive organizational climate, supporting, authorizing, positively evaluating, and crediting leaders who accept the training to develop PsyCap, which can give rise to expected outcomes (Petersen, 2015). Secondly, leaders should foster the effective transmission of their PsyCap. Besides this, leaders should not only transmit high PsyCap but also make efforts to transmit their PsyCap to all the members consistently, which can generate effective incentive to their teams (Rego et al., 2017). In other words, to make good use of leader PsyCap, leaders have to dedicate to building a fair and harmonious climate, present their passion, confidence, resilience, and creativity to their followers indifferently, be liable for their commitment, and keep a good interpersonal relation with their followers in the routine work.

Enterprises should emphasize and strengthen employee GNS. Although leader PsyCap can stimulate innovative behavior in employees, the effect can vary between employees with different levels of GNS. Because employees with high GNS exhibit more innovative behaviors when they perceive sufficient psychological safety, organizations can recruit such employees by designing specific standards for talent recruitment, thereby increasing innovative behavior. Additionally, enterprises can implement suitable training and propose corresponding encouragement policies to raise awareness of self-growth among employees. Subsequently, employees should be assigned suitable tasks at different stages and be provided with opportunities and a platform to fulfill their growth needs. Employees can then continually increase their growth needs, creating a virtuous cycle in which employees enhance their innovative thinking skills and develop the habit of innovation.

### Research Limitations and Future Research Directions

First, this study adopted an indirect measurement research method and collected data by using a questionnaire. Future research should adopt a quasi-experimental design or conduct experiments to enhance the accuracy of research conclusions. Second, this study used subjective indicators to measure the variables of interest. Third, this study did not take the sectors that leaders and subordinates were employed into account in the process of data analysis, although it is theoretically evidenced that GNS is highly correlated to the individuals’ working environment. Thus, future studies should incorporate the sectors that leaders and subordinates were employed into relevant statistical analysis. Currently, leader PsyCap research has been inadequate. In particular, research on the structure and measurement of leader PsyCap in China remains lacking and warrants exploration. Finally, although this study investigated the effect of leader PsyCap on innovative behavior mediated by psychological safety from the perspectives of employee awareness, motivation, and demands, several internal and external mechanisms in the research process might have remained unknown. Therefore, future studies should focus on clarifying these mechanisms.

## Data Availability Statement

The raw data supporting the conclusions of this article will be made available by the authors, without undue reservation.

## Author Contributions

YW designed the study and collected the data. YC wrote and revised the manuscript. YW and YZ gave guidance throughout the whole research process. All authors contributed to the article and approved the submitted version.

### Conflict of Interest

The authors declare that the research was conducted in the absence of any commercial or financial relationships that could be construed as a potential conflict of interest.
